# Enhancing Grapevine Node Detection to Support Pruning Automation: Leveraging State-of-the-Art YOLO Detection Models for 2D Image Analysis

**DOI:** 10.3390/s24216774

**Published:** 2024-10-22

**Authors:** Francisco Oliveira, Daniel Queirós da Silva, Vítor Filipe, Tatiana Martins Pinho, Mário Cunha, José Boaventura Cunha, Filipe Neves dos Santos

**Affiliations:** 1 School of Science and Technology, University of Trás-os-Montes and Alto Douro (UTAD), 5000-801 Vila Real, Portugal; vfilipe@utad.pt (V.F.); jboavent@utad.pt (J.B.C.); 2 INESC Technology and Science (INESC TEC), 4200-465 Porto, Portugal; daniel.q.silva@inesctec.pt (D.Q.d.S.); tatiana.m.pinho@inesctec.pt (T.M.P.); mccunha@fc.up.pt (M.C.); filipe.n.santos@inesctec.pt (F.N.d.S.); 3 Faculty of Sciences, University of Porto (FCUP), 4169-007 Porto, Portugal

**Keywords:** deep learning, precision agriculture, pruning, robotic systems, YOLO

## Abstract

Automating pruning tasks entails overcoming several challenges, encompassing not only robotic manipulation but also environment perception and detection. To achieve efficient pruning, robotic systems must accurately identify the correct cutting points. A possible method to define these points is to choose the cutting location based on the number of nodes present on the targeted cane. For this purpose, in grapevine pruning, it is required to correctly identify the nodes present on the primary canes of the grapevines. In this paper, a novel method of node detection in grapevines is proposed with four distinct state-of-the-art versions of the YOLO detection model: YOLOv7, YOLOv8, YOLOv9 and YOLOv10. These models were trained on a public dataset with images containing artificial backgrounds and afterwards validated on different cultivars of grapevines from two distinct Portuguese viticulture regions with cluttered backgrounds. This allowed us to evaluate the robustness of the algorithms on the detection of nodes in diverse environments, compare the performance of the YOLO models used, as well as create a publicly available dataset of grapevines obtained in Portuguese vineyards for node detection. Overall, all used models were capable of achieving correct node detection in images of grapevines from the three distinct datasets. Considering the trade-off between accuracy and inference speed, the YOLOv7 model demonstrated to be the most robust in detecting nodes in 2D images of grapevines, achieving F1-Score values between 70% and 86.5% with inference times of around 89 ms for an input size of 1280 × 1280 px. Considering these results, this work contributes with an efficient approach for real-time node detection for further implementation on an autonomous robotic pruning system.

## 1. Introduction

Pruning is a labour-intensive and costly agricultural task, which is mainly dependable on manual labour [1] and requires approximately between 70 and 90 h of work per ha according to the training system [2]. However, this task is highly relevant for certain species of plants, such as grapevines. Beyond mere aesthetics, pruning plays a crucial role in shaping plant health and growth by trimming branches [3]. Performing pruning on grapevines promotes the growth of healthier and stronger shoots, allowing for adjustments in crop-load while maintaining the vine’s balance [4]. This task is essentially performed in the winter, when the grapevines are dormant and leafless [5]. Due to the difficulty in having enough labour available to perform seasonal agricultural tasks, such as pruning, research on automating these tasks is increasingly becoming a topic of interest [6]. In the automation field, robotic pruning is a process that usually involves selective pruning of specific branches, similar to the method used manually [7]. To effectively deploy a robotic system capable of autonomously conducting pruning tasks, the system must possess comprehensive information on task completion procedures. This entails more than just providing the robot with visual guidance to navigate to the cutting point on the branch; it also requires endowing it with the ability to accurately select the appropriate location for the cut. In pruning, there are several rules and methods that can be followed to complete the procedure. Despite the pruning method employed, it is crucial to consider the canes’ nodes location when making the cutting decisions, since it is from those nodes that buds will grow new branches. Consequently, the number of buds left after pruning will influence the grapevine’s yield [8].

### 1.1. Related Work

Recently, there have been a few works focusing on algorithms both for branch and node detection to support agricultural task decisions, such as for pruning. Cuevas-Velasquez et al. [9] developed a cutting location algorithm focusing on completing pruning tasks on rose bushes. The authors’ approach was based on stem detection, making use of a Convolutional Neural Network (CNN) to segment the branches from the background of the image. The cutting point location was then selected according to the segmented stem’s height. Marset et al. [10] proposed the use of computer vision algorithms to detect buds in grapevine branches. For this purpose, the authors implemented a Fully Convolutional Network (FCN) with a MobileNet, which segmented pixels in the image corresponding to buds. Gentilhomme et al. [11] proposed a novel method for grapevine structure estimation to assist in pruning activities. The authors analysed the grapevines’ structure considering the similarities with the problem of human pose estimation, with the plant’s nodes as the landmarks and the branches as the spatial dependencies. Gentilhomme et al.’s method [11] consists essentially of a Stacked Hourglass Network (SHG), which outputs both the nodes and vector fields of the branches out of two-dimensional (2D) images of the grapevines. Afterwards, these features are associated according to the points’ coordinates, resulting in a complete vine structure.

### 1.2. Contributions

This paper aims to present efficient light-computing deep learning models for node detection on 2D images of grapevines. Despite the fact that recent works have been able to use 2D images to compute cutting locations for pruning tasks, they present constraints. In the work of Cuevas-Velasquez et al. [9], the cutting decision was based on the stems’ heights, not providing data on the detection of small objects such as nodes. The authors acknowledged that, in future work, the pruning criteria should be updated to consider more phenotypic traits. Meanwhile, in the research of Marset et al. [10], although a detection algorithm was implemented for bud detection in grapevines, the images used were close-ups of the branch, not allowing for visibility over the whole grapevine. The work of Gentilhomme et al. [11] successfully detected nodes in grapevines. However, the proposed network was trained with images containing an artificial background, and the authors considered that the algorithm was not robust enough to successfully perform on environments where the artificial background was removed from behind the grapevines.

This work aims to surpass the constraints present in the previous approaches for node detection by providing a solution that guarantees the following:

- Nodes are detected with the further objective of being considered for pruning rules;
- Node detection is achieved by the algorithm even when visualizing the entire grapevine;
- Elimination of artificial background requirement for the implementation of the detection model ensures acceptable accuracy, practicality, and versatility in real-world vineyard environments, where natural backgrounds vary widely;
- Further implementation on a real-time system is possible, not producing inference times that compromise the pruning task execution.

In order to achieve these requirements, this paper studies the adaptability of four distinct *You Only Look Once* (YOLO) [12] models (YOLOv7 [13], YOLOv8 [14], YOLOv9 [15] and YOLOv10 [16]) on performing node detection on grapevines. These models were trained with a public grapevine dataset containing images with artificial backgrounds and were further tested on detecting nodes of grapevines with distinct configurations on natural environments without artificial backgrounds, as detailed in Section 2.1.

Furthermore, this allowed us to understand the requirements to correctly detect the grapevine’s nodes on two distinct and highly relevant viticulture regions in Portugal, in which the grapevines’ configurations differ significantly, as well as the backgrounds created by the region’s landscapes and climate [17]. The work developed also resulted on the creation of a publicly available dataset of Portuguese grapevines for node detection [18], contributing as a first approach towards the development of a perception module for an autonomous robotic system capable of performing pruning tasks with equivalent or higher performance than the same type of task performed by human labour.

## 2. Materials and Methods

### 2.1. Datasets

In order to train the models, we used the publicly available 3D2cut Single Guyot dataset [11], whose respective authors created to develop a method for pruning task assistance. This choice was made mainly due to three reasons: the images captured contained the entire grapevine on each frame, making it possible to visually distinguish each constituent and to simultaneously perform the detection of the same plant on all the canes; a single grapevine was captured per frame in most of the images, avoiding the presence of additional unwanted information; the dataset was collected with an artificial background behind the grapevines, which resulted in a more intuitive annotation procedure, validation of the bounding boxes’ location and distinction of the different constituents of the plant.

**Figure 1:**
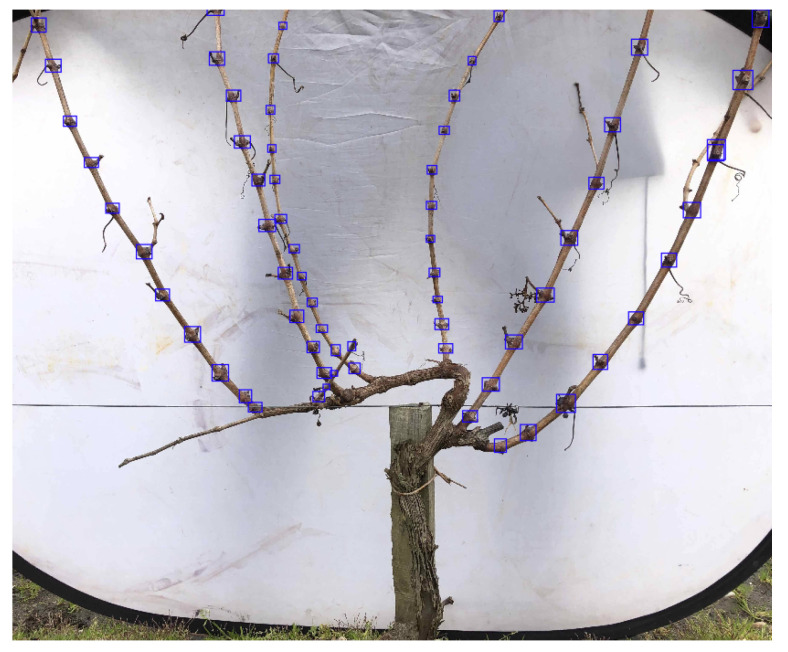
Manually annotated image from the 3D2cut Single Guyot dataset [11] containing only the nodes on the primary canes of the grapevine.

New annotations to the existing dataset have been performed to consider only the nodes on the primary canes of the grapevine in the centre of each image, which are the main detection goals since pruning will be conducted on these primary canes by considering the number of nodes present as a criterion. This step was performed to guarantee that it would achieve lightweight models for node detection by removing non-relevant information on the detection framework, such as nodes in areas of the grapevine not corresponding to primary canes. These annotations were performed using the Computer Vision Annotation Tool (CVAT) (CVAT. https://www.cvat.ai/ (accessed on 17 October 2024)) platform, with bounding boxes surrounding the portions of the canes containing the nodes, as seen in Figure 1.

To comprehensively assess the resilience of the deep learning models proposed in this study to varying grapevine conditions, particularly those with cluttered backgrounds, it became imperative to curate new datasets distinct from the 3D2cut Single Guyot dataset [11], mainly containing images without artificial backgrounds. Thus, images from two distinct Portuguese viticulture regions were used to improve this evaluation.

The first set of images was collected at *Centro de Estudos Vitivinícolas do Dão*, located in the *Dão* region (40°31′31.5″ N 7°51′22.7″ W), while the second dataset was obtained from *Quinta do Seixo* in the *Douro* region (41°10′05.4″ N 7°33′17.5″ W). The performance of the trained YOLO models on images from these two locations was considered relevant to this study because the grapevine configuration is different from the 3D2cut Single Guyot dataset [11]. Additionally, these images were captured without using artificial backgrounds on regions with distinct background environment information due to natural light sources and landscape, which might have influenced the outcome of the detection. Both datasets were acquired during the winter months, between December and January. The *Dão* dataset was captured on a clear afternoon with abundant light, whereas the *Douro* dataset was captured on a cloudy afternoon, where, despite sufficient lighting, the sunlight was less intense when compared to the *Dão* location.

**Figure 2:**
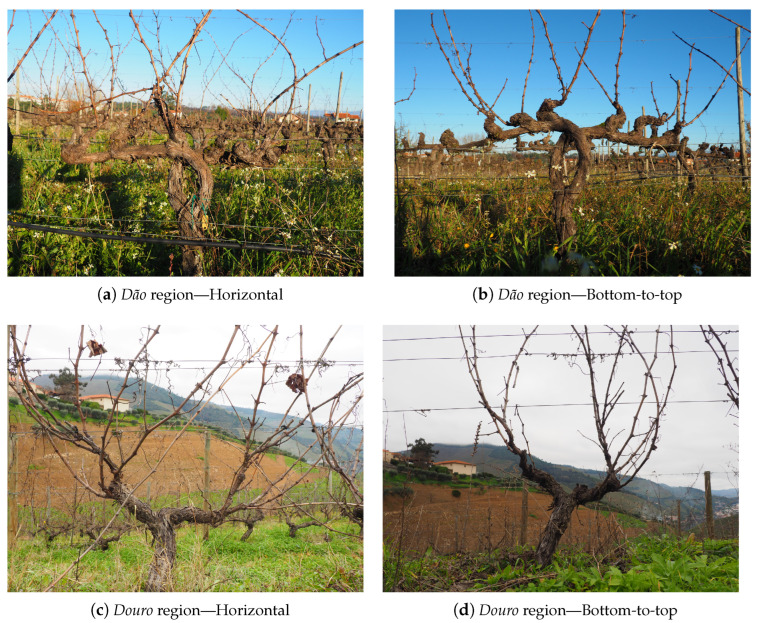
Examples of different perspectives of the captured images.

For both datasets, two camera perspective variations in relation to the grapevine were employed for further performance comparison. The first was a horizontal perspective, which allowed the background of the image to have more objects in sight, such as adjacent vineyard rows and hills. The second was a bottom-to-top perspective using the sky as a more uniform background of the image, as illustrated in Figure 2. The images were acquired using a digital OM System camera (OM System. https://explore.omsystem.com/us/en/ (accessed on 17 October 2024)) at a distance of approximately 80 cm of the captured grapevine with a resolution of 4608 × 3456 pixels (px).

### 2.2. Deep Learning Models

YOLO deep learning models have been widely used in real-time object detection and applied in various fields, including robotics [19]. These models are single-stage objective detection algorithms, granting them high speed combined with precision [20]. Due to the detection performance reported by their respective authors on the Microsoft Common Objects in Context (MS COCO) dataset [21], the following state-of-the-art YOLO versions were selected for this work.

YOLOv7 was published in 2022 and at the time surpassed the previously developed object detectors in terms of speed and accuracy. This accuracy was improved without incrementing the inference speed at the cost of increased training time [22]. The authors also designed a version of this model to be run in edge devices—the YOLOv7-tiny [13]—and since the goal of this work was to study detection algorithms capable of real-time inference on an autonomous robotic system, this version was chosen as one of the models for this purpose.

The YOLOv8 is one of the most recent object detection models, released in 2023. It has a backbone similar to the one used on YOLOv5 [23] with modifications that allow for improvements on detection accuracy [22]. Moreover, the YOLOv8 uses Distribution Focal Loss (DFL) [24] and Complete Intersection over Union (CIoU) [25] loss functions for the bounding-box loss, which is expected to contribute to improving the object detection performance in smaller objects [22], as it is relevant for node detection scenarios due to the size of the nodes when compared to the full image of a grapevine. In order to guarantee that the model used could compute light and was capable of being implemented on a real-time object detection system, the model selected was the YOLOv8 Small (YOLOv8s), which is the second-smallest scaled version of the model provided by Ultralytics (Ultralytics. https://www.ultralytics.com/ (accessed on 17 October 2024)). Furthermore, previous works have already developed on bud detection based on this version of the YOLOv8 model. Xie et al. [20] implemented the YOLOv8s to detect buds on tea trees, achieving a Mean Average Precision (mAP) of 88.27% and an inference time of 37.1 ms.

In 2024, the YOLOv9 was published by the same authors of the YOLOv7. The authors designed a novel lightweight network architecture called the Generalized Efficient Layer Aggregation Network (GELAN), which, when combined with the concept of Programmable Gradient Information (PGI), also developed by the same authors, allowed it to surpass the performance of the existing object detectors. The PGI framework on the GELAN architecture helped to reduce information bottleneck during the feedforward process, which resulted in more retained complete information when compared to architectures such as PlainNet, ResNet and CSPNet [15]. In order to compare the performance of this novel YOLO model in grapevine node detection with the previously presented ones, we used the light-computing YOLOv9 Small (YOLOv9-S).

Also in 2024, YOLOv10 was announced with a novel approach for real-time object detection, eliminating the use of non-maximum suppression (NMS). The model is based on the YOLOv8 from Ultralytics, which the authors have chosen due to the efficient balance achieved between latency and accuracy. Despite being based on the YOLOv8, according to the authors, the YOLOv10 achieves higher average precision (AP) while requiring less parameters and fewer calculations and also achieving lower latencies. The YOLOv10 was developed containing dual-label assignments, which, during the training phases, recurs to two heads to optimize the model. However, during inference, only one head is maintained (one-to-one) to make predictions, avoiding using NMS in post-processing. Similarly to the previous ones, the model used was the small variant: the YOLOv10 Small (YOLOv10-S).

### 2.3. Training Configuration and Augmentations

The four distinct YOLO models presented were trained on a total of 13,539 images of grapevines, whereby 759 of the images were from the 3D2cut Single Guyot dataset [11] and the remaining 12,780 images were from augmentations of these original images.

**Figure 3:**
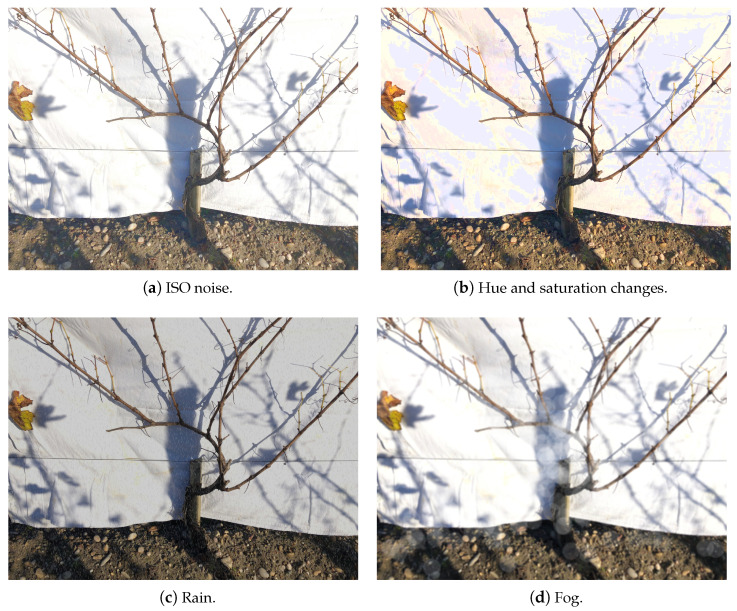
Examples of augmentations performed on the 3D2cut Single Guyot dataset [11] in order to increase the variability of the dataset.

The 3D2cut Single Guyot dataset [11] contained varied images of grapevines with significant differences between vines not only on their main structure, but also on cane orientation and size. However, this dataset is not a complete representation of possible environments and scenarios in a real-life application, lacking variations in the image capture perspective as well as external elements that might be present in the field. To address the lack of variability in this dataset, the following augmentations presented in Table 1 were implemented. These augmentations used to improve the training image set presented geometric modifications to the existing dataset (image flip, scaling, and angle change), and also inserted some external elements that can be present in real-life applications on the field, such as rain, mud, fog, blur, variations in hue and saturation, ISO noise, and optical distortion, as can be seen in Figure 3. These altered images allowed us to transform the dataset to a more reliable approximation of the agricultural work environment, which is often unpredictable and subjected to harsh and variable climatic conditions [26,27].

The deep learning models used were initially trained with similar hyperparameters for 50 epochs. However, there was a lack of convergence on the training loss for all the models. Therefore, the number of training epochs was gradually incremented to 150 for all the models to guarantee that the training loss value stabilized. Despite using similar hyperparameters in the first set of training conducted, eventually, a few suffered different tuning strategies to guarantee that the best results from each model were achieved as well as to ensure correct adaptation to the hardware used. Parameters such as input and batch sizes had to be modified on all models due to constraints on the hardware of the device used for training (NVIDIA GeForce RTX 3090, NVIDIA Corporation, Santa Clara, CA, USA) (NVIDIA GeForce RTX 3090. https://www.nvidia.com/en-eu/geforce/graphics-cards/30-series/rtx-3090-3090ti/ (accessed on 17 October 2024)). However, YOLOv8s, YOLOv9-S and YOLOv10-S presented more difficulties to reach convergence during training. For this purpose, the Cosine learning Rate Scheduler Function [28] was activated during training, and mosaic data augmentation was deactivated on the last 50 epochs to improve loss stabilization at the last training stages. Furthermore, different optimizers were tested on different trainings, and it was discovered that on all the models tested, the AdamW contributed to better convergence. The basic configurations of the models and the hyperparameters that suffered further modifications in each deep learning model used, which resulted in the most favourable results, can be seen in Table 2.

## 3. Results

**Figure 4:**
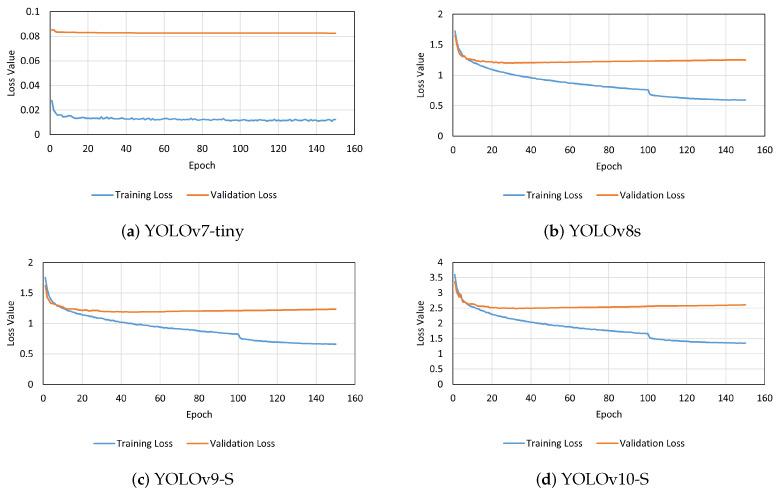
Box loss values on the four models trained.

The trained YOLO models demonstrated convergence within the 150 epochs of training. However, the YOLOv7-tiny resulted in smaller box loss values for both training and validation, and those values were more stable at the end of the training when compared to the other models. The other models started to present a slight increase in the validation loss, which can be an indicator that the model was starting to overfit [29]. In contrast, YOLOv10-S exhibited higher loss values during training compared to the other models. The box loss values of the trained models are presented in Figure 4.

### Validation and Field Trials

Three different sets of images were selected to evaluate the performance of the models in detecting the nodes on grapevines. The first set was composed of images from the 3D2cut Single Guyot dataset [11], containing an artificial background behind the captured grapevines. The remaining two sets consisted of images selected from the datasets gathered on the Portuguese *Dão* and *Douro* regions, containing 30 original images and 150 augmented images, which added geometric modifications to the original images in order to increase the variability of the dataset by containing grapevines captured in different layouts.

The images from the 3D2cut Single Guyot dataset [11] were used as a benchmark to compare the robustness of the YOLO models trained. Since these images were similar to the ones used for training, it was expected that the models would achieve the best results. *Dão* and *Douro* datasets constituted of images taken in open fields without covering the objects behind the grapevines. This feature allowed these datasets to be used as indicators to evaluate the robustness of the models developed in detecting nodes in images with natural backgrounds (cluttered).

Furthermore, despite the fact that the models have been trained with an input size of 640 × 640 px, tests were also conducted with changes in the input parameter to consider a size of 1280 × 1280 px. Since nodes on grapevines are relatively small objects on the overall size of the image, this test was crucial to reduce the loss of information on the images used. However, it was expected that this increase in the input size would result in delays in the inference times of the models [30].

The results obtained on the validation phase of the detection models trained on the four different datasets are presented in Table 3. The metrics obtained were analysed at confidence levels of at least 10% and at the best F1-Score obtained in each model. Confidence levels below 10% were excluded from the analysis, as lower confidence scores are likely to lead to an increase in false positive detections [31]. An increase in false positives would negatively affect the performance of the node detection task, resulting in inaccurate conclusions and potentially compromising the reliability of the system to be integrated into an autonomous pruning robot. Furthermore, Table 4 displays the average inference time per image on each of the models with different input sizes.

## 4. Discussion

Overall, YOLOv7-tiny achieved higher F1-Score values than the other models. The more recent models tested, despite typically presenting slightly higher Precision values, resulted in lower Recall values. Moreover, the mAP obtained by the novel YOLOv10-S was usually significantly lower than the other models benchmarked for scenarios significantly different from the training environment.

**Figure 5:**
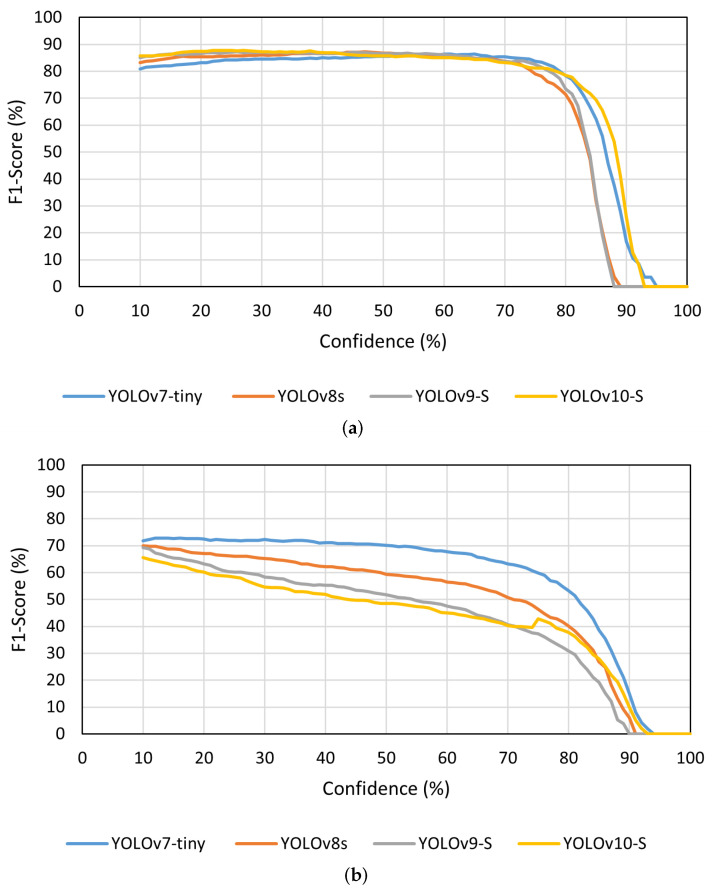

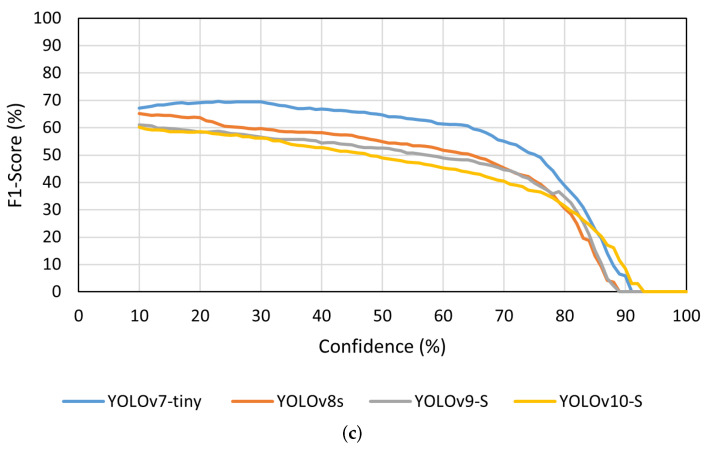
F1-Score of the models with an input size of 1280 × 1280 px in the three datasets used. (**a**) 3D2cut Single Guyot [11]; (**b**) *Dão*; (**c**) *Douro*.

Observing the results previously presented in Table 3, there was a tendency for the models to perform better with an input size of 1280 × 1280 px when compared to the standard input size of 640 × 640 px, which supports the possibility of information being lost in smaller images due to the reduced size of the nodes. Due to this characteristic, it was considered advantageous to use this larger input size on the developed models, which demonstrated performance improvements. Figure 5 shows a comparison of the F1-Score values from the four YOLO models analysed on the different datasets used, considering an input size of 1280 × 1280 px.

Despite these performance differences between the used versions, all the models were capable of detecting the grapevine’s nodes successfully. Furthermore, it was possible to observe that on the 3D2cut Single Guyot dataset [11], the four models achieved F1-Score values of above 80%, despite the input size used. Meanwhile, on both *Dão* and *Douro* datasets, the highest F1-Score values were around 70%. However, since the objective of this study is to further implement a perception algorithm on a robotic system capable of performing pruning tasks autonomously, not only is it crucial to analyse the models’ accuracy and their adaptability to different real-case scenarios, but also how much time they take to complete the inference.

### 4.1. Adaptability to Different Grapevine’s Configurations and Environments

**Figure 6:**
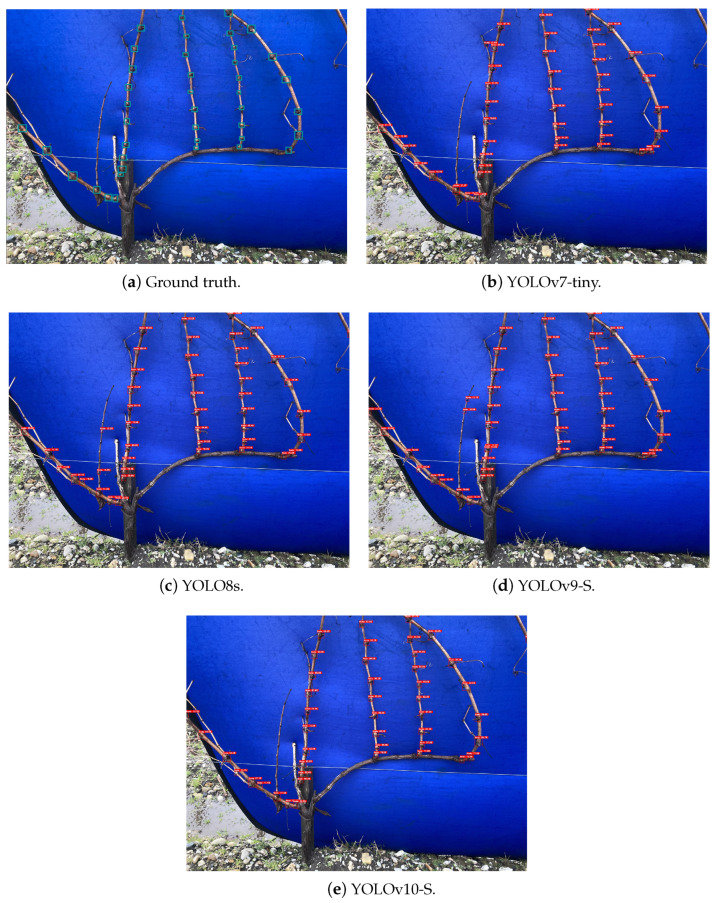
Inference on a grapevine from the 3D2cut Single Guyot dataset [11] considering an input size of 1280 × 1280 px.

As expected, the models’ inference performance was considerably higher on the 3D2cut Single Guyot dataset [11], where all the models presented a similar detection capability, as seen in Figure 6. This behaviour occurs not only because this set constituted of images similar to the ones used for training, but also due to the amount of additional information on the background of the *Dão* and *Douro* datasets, which induced the occurrence of some false positive detections on the background of the images.

In both of these two datasets (*Dão* and *Douro*), we noticed that the YOLOv7-tiny achieved higher F1-Score values than the other three models, with this difference being more noticeable on these datasets in comparison to the results obtained on the 3D2cut Single Guyot dataset [11]. Furthermore, although YOLOv8s was the second best performing model on the *Dão* dataset, the performance changed significantly on the *Douro* dataset, in which this model presented varying results according to the input size used, resulting in lower F1-Score values for an input size of 640 × 640 px. Although the Precision of YOLOv10-S was usually higher than the other models on the Portuguese datasets, the Recall was lower, especially when considering an input size of 640 × 640 px. This allowed us to notice that when using the YOLOv10-S, despite not encountering many false positives, the model presented more difficulties in detecting small objects on each frame, ignoring a few nodes in the image. This behaviour makes it less valuable for the intended task than the other models analysed, since this behaviour results in a considerable amount of nodes not being considered as such.

**Figure 7:**
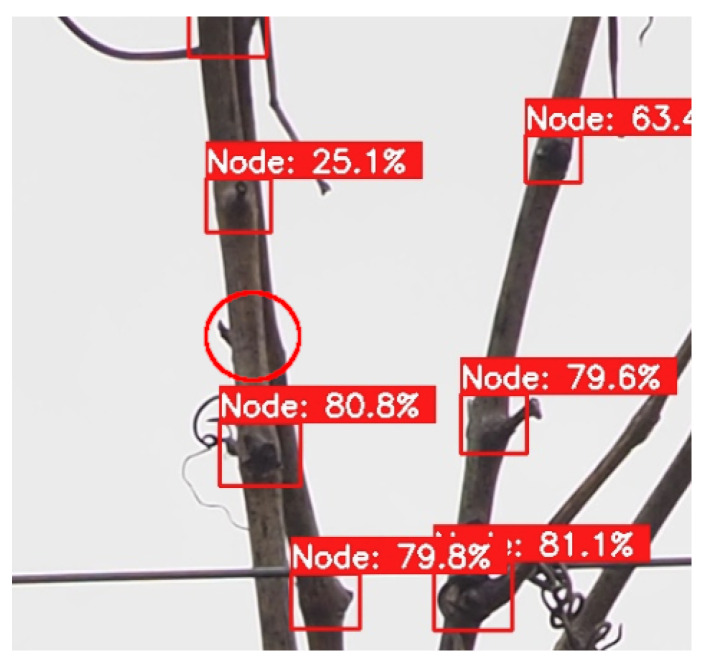
Partially occluded node (red circle) not detected in image from *Douro* dataset.

Furthermore, when comparing the results obtained between the two datasets without an artificial background, the *Douro* dataset resulted in less-accurate detections by the models than the *Dão* dataset. Analysing the images present in each one, it is possible to observe what features might be causing this discrepancy. The grapevines on the *Dão* dataset, despite having a structure different from the grapevines on the images of the training dataset, make distinguishing each cane on the plant easily feasible. Meanwhile, on the *Douro* dataset, the grapevines have more canes overlapped, and thus sometimes make it more difficult to distinguish the primary canes; this also results in nodes that can be occluded by nearby branches, as can be observed in Figure 7.

**Figure 8:**
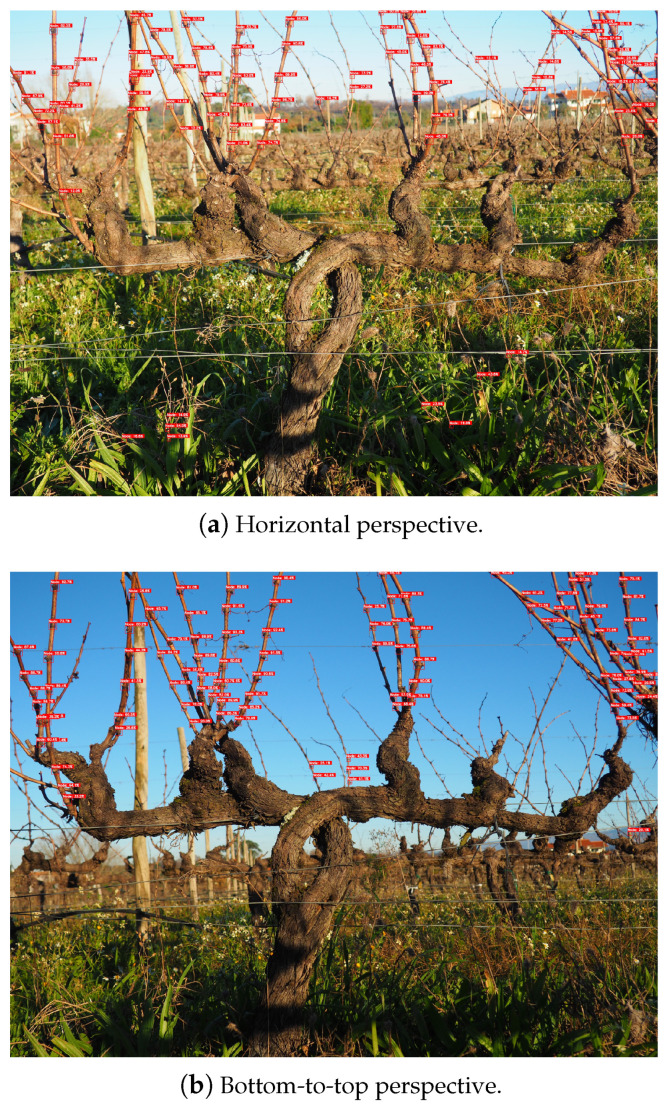
YOLOv7 inference comparison considering two different camera perspectives.

Regarding the annotations performed on the 3D2cut Single Guyot dataset [11], only the nodes on the primary canes of the centred grapevine on the image were selected. The same logic was implemented on the construction of the ground truth for the *Dão* and *Douro* image sets. However, the models also detected a few nodes on canes in adjacent grapevines that slightly appeared on the images captured. This consequently resulted in these detections being considered as false positives, which is not entirely wrong since the nodes do not belong to the focused grapevine, but they are correctly assumed as being nodes. Although these situations slightly affect some of the images, they were not considered relevant for the overall performance of the models in detecting nodes on these datasets, since it only happened in a few cases. Furthermore, in these sets of images taken in open field, the pictures captured in a bottom-to-top perspective are preferable to achieve better results. This approach helped reduce the background information, resulting in less false positives detected. In Figure 8, it is possible to notice that a horizontal camera perspective captures additional information, not only from other grapevines behind the one in foreground, but also from the ground of the figure. Meanwhile, a bottom-to-top perspective reduces the amount of content in the background of the image, allowing for a more precise detection.

Regardless of the performance differences between datasets and between images containing artificial backgrounds or not, grapevine node detection recurring to YOLO detection models presents an efficient and more versatile approach when compared to the previously implemented methods. The previously published work by Gentilhomme et al. [11] managed to achieve an overall Precision of 95% and Recall of 90% in the task of node detection, having an execution time between 0.55 s and 8 s. Despite obtaining good detection performance on the selected testing environment, the authors acknowledged that the algorithm requires optimization to be implemented on a real-time system and also considered that the algorithm proposed for pruning assistance would be severely impacted in work conditions without uniform artificial backgrounds. However, this work assures that grapevine node detection is feasible in heterogeneous environments recurring to state-of-the-art YOLO detection models, despite presenting a slight performance decrease.

**Figure 9:**
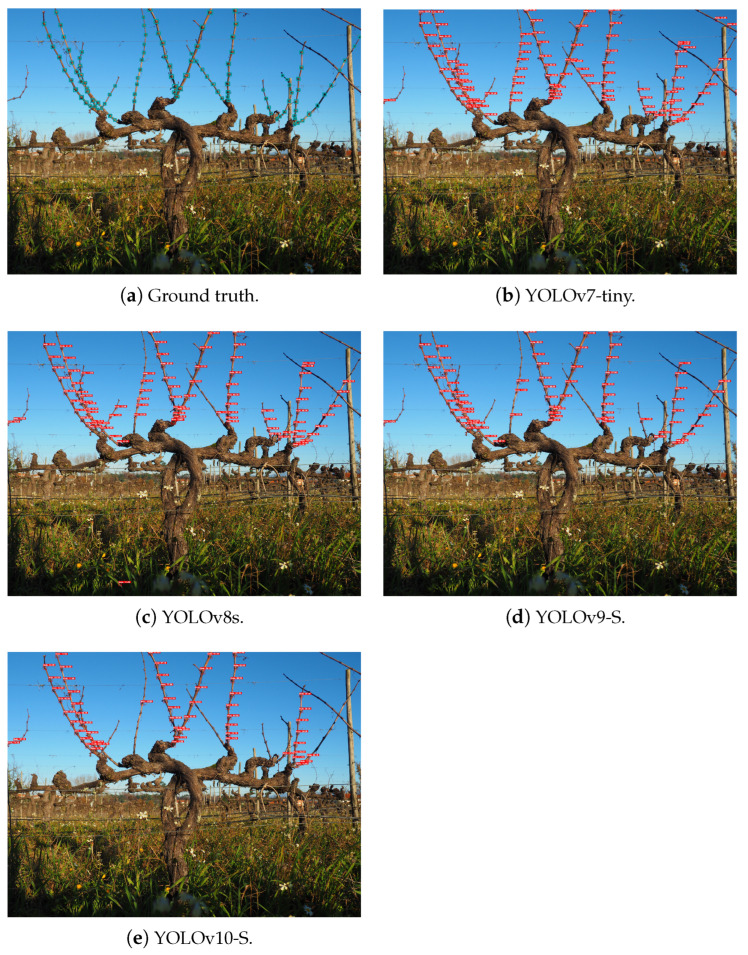
Inference on grapevines from the *Dão* region.

**Figure 10:**
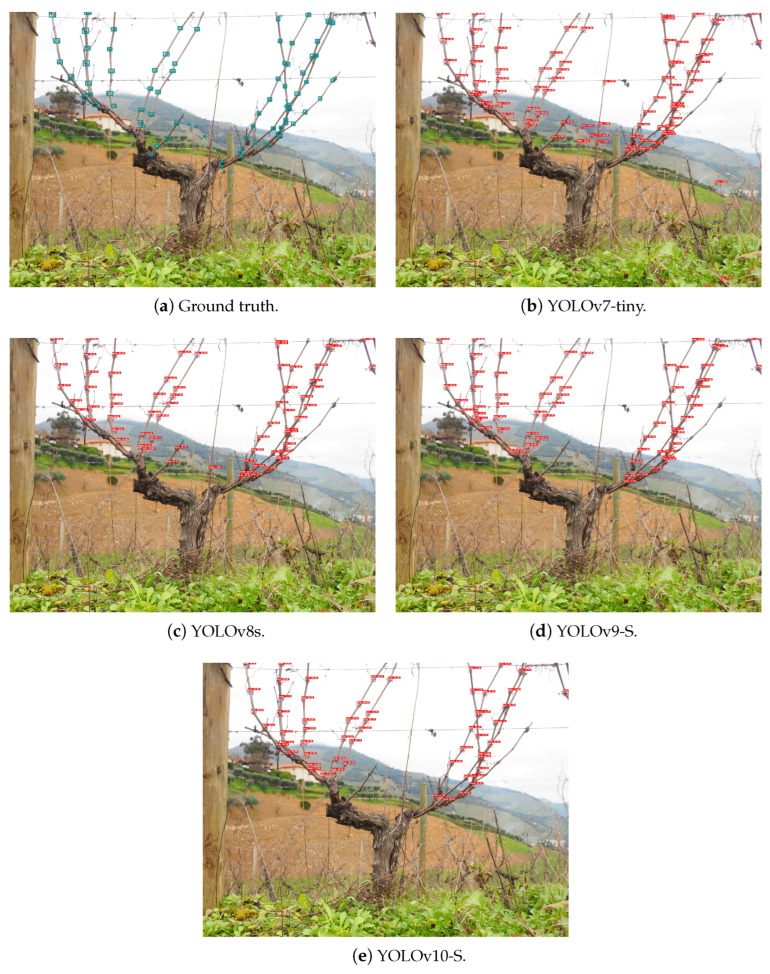
Inference on grapevines from the *Douro* region.

Furthermore, it was possible to assess the capability of performing node detection on the entirety of the grapevines even when configurations different from those used in training are provided to the models. In Figure 9 and Figure 10, it is possible to analyse the inference behaviour of the models used on each of the datasets acquired in Portugal, *Dão* and Douro, respectively, considering an input size of 1280 × 1280 px.

### 4.2. Capability of Implementation in a Real-Time System

According to the work by Botterill et al. [1], a human takes approximately 2 min to prune a grapevine. In a robotic system, there might be several necessary additional steps from the detection of the cutting location until the cut is completed. These steps are mainly related to navigation towards the cutting location and actuation of the cutting tool [7,32]. Considering this, minimizing the inference time of the detection algorithm is crucial to ensure the maximum efficiency of the system by reducing the necessary time to execute the pruning task on each vine.

The average inference time of the analysed YOLO models varied significantly. These inference time differences were not only between the distinct versions of the models, but also between different input sizes. YOLOv7-tiny was the fastest model at grapevine node detection, presenting inference times below 100 ms. YOLOv8s, YOLOv9-S and YOLOv10-S presented significantly larger times, with YOLOv8s reaching an average of around 502.5 ms when detecting nodes with a 1280 × 1280 px input size.

Despite the most recent models (YOLOv9-S and YOLOv10-S) being faster than the 502 ms of YOLOv8s, their inference times on 1280 × 1280 px sized images are still near 300 ms, which is significantly higher than the inference time obtained using the YOLOv7-tiny. Furthermore, the YOLOv7-tiny model presents less computational complexity than the other models tested, having 6.2 million (M) parameters and 13.8 Giga (G) Floating Point Operations Per Second (FLOPS). This number of operations corresponds to around 36% fewer operations than YOLOv10-S, which is the second model tested with fewer FLOPS, as can be seen in Table 5. Considering this, as a matter of the capability of implementation in a real-time system, the YOLOv7-tiny would be the preferred model of the four in order to ensure the system’s efficiency.

## 5. Conclusions

This work allowed us to compare, side-by-side, four distinct YOLO models on the capability of successfully performing the task of node detection of a grapevine’s canes. The models were trained on the publicly available 3D2cut Single Guyot dataset, which contained an artificial background on the captured images. Furthermore, the models were tested on two different Portuguese grapevine datasets without artificial backgrounds, which allowed us to evaluate the robustness of the models on two distinct cultivars with different surrounding environments.

Considering this approach, it was possible to notice that YOLOv7-tiny achieved the best detection performance compared to the other used models. Furthermore, YOLOv7-tiny was the best model considering the trade-off between accuracy and inference speed. This detection model not only achieved overall better metrics compared to the state-of-the-art detection models, but also presented the fastest inference speed for both tested input sizes (640 × 640 px and 1280 × 1280 px).

Despite the significant performance variation between solutions, all the state-of-the-art YOLO models used are capable of detecting the nodes on grapevines even when additional environment information is present on the background of the frames behind the vines, which is an improvement to previously developed algorithms that lacked robustness to actuate in heterogeneous environments. This approach of node detection recurring to YOLO detection models fulfilled the objectives of presenting efficient and light-computing deep learning models for node detection on 2D images of grapevines. These models are capable of detecting nodes in cluttered environments while visualizing the entire grapevine, and they offer inference times suitable for real-time system implementation. Based on these features, the models demonstrate sufficient robustness for integration with decision-making algorithms to predict potential pruning locations on grapevine canes, even in highly heterogeneous settings. Notably, this performance is achieved without the need for artificial backgrounds to reduce background interference. Additionally, the low inference times, particularly for YOLOv7-tiny, enable the practical deployment of these algorithms on an autonomous pruning robot, allowing real-time operation without compromising system efficiency.

However, despite being proven the feasibility of implementing YOLO-detecting models on these types of environments, as future work, it would be interesting to perform the detections by firstly filtering the background of the images using data from a depth camera to remove unwanted information, which is expected to also improve inference times since the information available for inference will be reduced.

## Figures and Tables

**Table 1:** Augmentation operations implemented.

Augmentation Operation	Values	Description
Horizontal Flip	-	Flips the image horizontally
Scale	0.7×	Scales down the image by 30%
	1.3×	Scales up the image by 30%
Rotation	−15°	Rotates the image −15°
	+15°	Rotates the image 15°
Hue, Saturation and Value	−15 ≤ *hue* ≤ 1	Changes the image’s hue, saturation and value levels
	−20 ≤ *saturation* ≤ 20	
	−30 ≤ *value* ≤ 30	
CLAHE	*contrast limit* = 4	Applies Contrast Limited Adaptive Histogram Equalization to the image
	*grid size* = 8 × 8	
Emboss	0.4 ≤ *alpha* ≤ 0.6	Embosses the image and overlays the result with the original image
	0.5 ≤ *strength* ≤ 1.5	
Sharpen	0.2 ≤ *alpha* ≤ 0.5	Sharpens the image and overlays the result with the original image
	0.5 ≤ *lightness* ≤ 1.5	
Optical Distortion	−0.15	Applies negative optical distortion to the image
	+0.15	Applies positive optical distortion to the image
Gaussian Blur	*blur* ≤ 7	Blurs the image using a Gaussian filter
	σ ≤ 5	
Glass Blur	σ = 0.5	Applies glass blur to the image
	δ = 2	
ISO Noise	*colour shift* = 0.15	Applies ISO noise to the image
	*intensity* = 0.6	
Random Rain	-	Adds random rain to the image
Random Fog	-	Adds random fog to the image
Random Snow	-	Adds random snow to the image
Spatter Mud	-	Adds mud spatter to the image
Spatter Rain	-	Adds rain spatter to the image

**Table 2:** Tuned hyperparameters used in each YOLO model.

Parameter	YOLOv7-tiny	YOLOv8s	YOLOv9-S	YOLOv10-S
Input Size	640 × 640 px	640 × 640 px	640 × 640 px	640 × 640 px
Batch Size	16	16	16	16
Initial Learning Rate	0.01	0.01	0.01	0.01
Final Learning Rate	0.0002	0.0002	0.0002	0.002
Optimizer	AdamW	AdamW	AdamW	AdamW
Cos_lr Function	False	True	True	True
Cls_mosaic Parameter	-	50	50	50

**Table 3:** Performance of the models in images from the different datasets.

Dataset	Model	Input Size	Precision	Recall	F1-Score	mAP@50	Precision	Recall	F1-Score	mAP@50	Average
		(px)	Confidence ≥ 10%				On the Best F1-Score				IoU
3D2cut	YOLOv7-tiny	640 × 640	84.5%	**86.8%**	**85.6%**	**83.4%**	84.5%	**86.8%**	**85.6%**	**83.4%**	78.6%
	YOLOv8s		90.9%	73.8%	81.5%	71.3%	90.9%	73.8%	81.5%	71.3%	80.5%
	YOLOv9-S		**91.1%**	76.2%	83%	74.6%	**91.1%**	76.2%	83%	74.6%	**80.9%**
	YOLOv10-S		87.9%	77.3%	82.3%	75.3%	87.9%	77.3%	82.3%	75.3%	80.6%
	YOLOv7-tiny	1280 × 1280	71.8%	92.6%	80.9%	86.6%	**88.8%**	84.3%	86.5%	80.1%	76.3%
	YOLOv8s		76.3%	91.4%	83.2%	84.9%	87.6%	86.8%	87.2%	80.8%	77%
	YOLOv9-S		78.4%	**93.1%**	85.1%	**88.5%**	84.1%	**90.5%**	87.2%	**86.1%**	78.3%
	YOLOv10-S		**80.6%**	91.4%	**85.7%**	86.8%	85.8%	89.8%	**87.8%**	85.2%	**79.1%**
*Dão*	YOLOv7-tiny	640 × 640	79%	**49.5%**	**60.8%**	**44.5%**	79%	**49.5%**	**60.8%**	**44.5%**	**70.8%**
	YOLOv8s		**85.5%**	19%	31.1%	18.8%	**85.5%**	19%	31.1%	18.8%	69.6%
	YOLOv9-S		76.6%	16%	26.4%	13.9%	76.6%	16%	26.4%	13.9%	63.2%
	YOLOv10-S		78.9%	13.9%	23.7%	12%	78.9%	13.9%	23.7%	12%	67.2%
	YOLOv7-tiny	1280 × 1280	73.4%	**70.4%**	**71.8%**	**61.5%**	76.4%	**69.7%**	**72.9%**	**60.8%**	**74.8%**
	YOLOv8s		76.4%	64.7%	70%	53.9%	76.4%	64.7%	70%	53.9%	73.6%
	YOLOv9-S		79.3%	61.4%	69.2%	50.8%	79.3%	61.4%	69.2%	50.8%	72.2%
	YOLOv10-S		**80%**	55.4%	65.5%	46.1%	**80%**	55.4%	65.5%	46.1%	73.3%
*Douro*	YOLOv7-tiny	640 × 640	**72.2%**	**47%**	**56.9%**	**42%**	**72.2%**	**47%**	**56.9%**	**42%**	**66.5%**
	YOLOv8s		67.3%	18.7%	29.2%	14.6%	67.3%	18.7%	29.2%	14.6%	61.7%
	YOLOv9-S		70.6%	19.9%	31%	15.9%	70.6%	19.9%	31%	15.8%	64.1%
	YOLOv10-S		68.5%	18.9%	29.6%	14.5%	68.5%	18.9%	29.6%	14.5%	62.4%
	YOLOv7-tiny	1280 × 1280	63.9%	**70.6%**	**67.1%**	**62.8%**	74.2%	**65.5%**	**69.6%**	**59.3%**	68.5%
	YOLOv8s		72.7%	58.9%	65.1%	52%	72.7%	58.9%	65.1%	52%	68.2%
	YOLOv9-S		71.1%	53.5%	61.1%	46.4%	71.1%	53.5%	61.1%	46.4%	67%
	YOLOv10-S		**77.4%**	49.2%	60.2%	44.9%	**77.4%**	49.2%	60.2%	44.9%	**69.2%**

Text in **Bold** indicates the best-performing results for each metric.

**Table 4:** Average inference time of the YOLO models.

Model	Input Size (px)	Average Inference Time (ms)
YOLOv7-tiny	640 × 640	**20.52**
YOLOv8s		323.79
YOLOv9-S		63.82
YOLOv10-S		51.59
YOLOv7-tiny	1280 × 1280	**88.79**
YOLOv8s		502.52
YOLOv9-S		288.53
YOLOv10-S		260.21

Text in **Bold** indicates the shortest inference time for each input size.

**Table 5:** Complexity comparison of the models tested.

Model	Number of Parameters	FLOPS
YOLOv7-tiny	6.2 M	13.8 G
YOLOv8s	11.2 M	28.6 G
YOLOv9-S	7.1 M	26.4 G
YOLOv10-S	7.2 M	21.6 G

## Data Availability

The data presented in this study are openly available in the digital repository Zenodo: Douro & Dão Grapevines Dataset for Node Detection—https://doi.org/10.5281/zenodo.10991688.

## Abbreviations

The following abbreviations are used in this manuscript:

2D	Two-dimensional
AP	Average Precision
CIoU	Complete Intersection over Union
CNN	Convolutional Neural Network
CVAT	Computer Vision Annotation Tool
DFL	Distribution Focal Loss
FCN	Fully Convolutional Network
FLOPS	Floating Point Operations Per Second
GELAN	Generalized Efficient Layer Aggregation Network
mAP	Mean Average Precision
MS COCO	Microsoft Common Objects in Context
NMS	Non-Maximum Suppression
PGI	Programmable Gradient Information
SHG	Stacked Hourglass Network
YOLO	You Only Look Once

## References

[1] Botterill T., Paulin S., Green R.D., Williams S., Lin J., Saxton V., Mills S., Chen X., Corbett-Davies S. (2017). A Robot System for Pruning Grape Vines. J. Field Robot..

[2] Poni S., Sabbatini P., Palliotti A. (2022). Facing Spring Frost Damage in Grapevine: Recent Developments and the Role of Delayed Winter Pruning—A Review. Am. J. Enol. Vitic..

[3] Reich L. (2010). The Pruning Book.

[4] Silwal A., Yandun F., Nellithimaru A., Bates T., Kantor G. (2021). Bumblebee: A Path Towards Fully Autonomous Robotic Vine Pruning. arXiv.

[5] Williams H., Smith D., Shahabi J., Gee T., Nejati M., McGuinness B., Black K., Tobias J., Jangali R., Lim H. (2023). Modelling wine grapevines for autonomous robotic cane pruning. Biosyst. Eng..

[6] Oliveira F., Tinoco V., Magalhães S., Santos F.N., Silva M.F. End-Effectors for Harvesting Manipulators—State of the Art Review. Proceedings of the 2022 IEEE International Conference on Autonomous Robot Systems and Competitions (ICARSC).

[7] He L., Schupp J. (2018). Sensing and Automation in Pruning of Apple Trees: A Review. Agronomy.

[8] Collins C., Wang X., Lesefko S., De Bei R., Fuentes S. (2020). Effects of canopy management practices on grapevine bud fruitfulness. OENO ONE.

[9] Cuevas-Velasquez H., Gallego A.J., Tylecek R., Hemming J., Van Tuijl B., Mencarelli A., Fisher R.B. Real-time Stereo Visual Servoing for Rose Pruning with Robotic Arm. Proceedings of the 2020 IEEE International Conference on Robotics and Automation (ICRA).

[10] Villegas Marset W., Pérez D.S., Díaz C.A., Bromberg F. (2021). Towards practical 2D grapevine bud detection with fully convolutional networks. Comput. Electron. Agric..

[11] Gentilhomme T., Villamizar M., Corre J., Odobez J.M. (2023). Towards smart pruning: ViNet, a deep-learning approach for grapevine structure estimation. Comput. Electron. Agric..

[12] Redmon J., Divvala S., Girshick R., Farhadi A. You Only Look Once: Unified, Real-Time Object Detection. Proceedings of the 2016 IEEE Conference on Computer Vision and Pattern Recognition (CVPR).

[13] Wang C., Bochkovskiy A., Liao H. YOLOv7: Trainable Bag-of-Freebies Sets New State-of-the-Art for Real-Time Object Detectors. Proceedings of the 2023 IEEE/CVF Conference on Computer Vision and Pattern Recognition (CVPR).

[14] Jocher G., Chaurasia A., Qiu J. (2023). Ultralytics YOLOv8. https://docs.ultralytics.com/pt/models/yolov8/.

[15] Wang C.Y., Liao H.Y.M. (2024). YOLOv9: Learning What You Want to Learn Using Programmable Gradient Information. arXiv.

[16] Wang A., Chen H., Liu L., Chen K., Lin Z., Han J., Ding G. (2024). YOLOv10: Real-Time End-to-End Object Detection. arXiv.

[17] Lavrador da Silva A., João Fernão-Pires M., Bianchi-de-Aguiar F. (2018). Portuguese vines and wines: Heritage, quality symbol, tourism asset. Ciência Téc. Vitiv..

[18] Oliveira F.A., Silva D.Q. (2024). Douro & Dão Grapevines Dataset for Node Detection.

[19] Casas G.G., Ismail Z.H., Limeira M.M.C., da Silva A.A.L., Leite H.G. (2023). Automatic Detection and Counting of Stacked Eucalypt Timber Using the YOLOv8 Model. Forests.

[20] Xie S., Sun H. (2023). Tea-YOLOv8s: A Tea Bud Detection Model Based on Deep Learning and Computer Vision. Sensors.

[21] Lin T.Y., Maire M., Belongie S., Bourdev L., Girshick R., Hays J., Perona P., Ramanan D., Zitnick C.L., Dollár P. (2015). Microsoft COCO: Common Objects in Context. arXiv.

[22] Terven J., Córdova-Esparza D.M., Romero-González J.A. (2023). A Comprehensive Review of YOLO Architectures in Computer Vision: From YOLOv1 to YOLOv8 and YOLO-NAS. Mach. Learn. Knowl. Extr..

[23] Jocher G. (2020). YOLOv5 by Ultralytics.

[24] Li X., Wang W., Wu L., Chen S., Hu X., Li J., Tang J., Yang J. Generalized focal loss: Learning qualified and distributed bounding boxes for dense object detection. Proceedings of the 34th International Conference on Neural Information Processing Systems.

[25] Zheng Z., Wang P., Liu W., Li J., Ye R., Ren D. (2020). Distance-IoU Loss: Faster and Better Learning for Bounding Box Regression. Proc. AAAI Conf. Artif. Intell..

[26] Vougioukas S.G. (2019). Agricultural Robotics. Annu. Rev. Control. Robot. Auton. Syst..

[27] Bechar A., Vigneault C. (2016). Agricultural robots for field operations: Concepts and components. Biosyst. Eng..

[28] Hsueh B.Y., Li W., Wu I.C. Stochastic Gradient Descent With Hyperbolic-Tangent Decay on Classification. Proceedings of the 2019 IEEE Winter Conference on Applications of Computer Vision (WACV).

[29] Prechelt L., Orr G.B., Müller K.R. (1998). Early Stopping—But When?. Neural Networks: Tricks of the Trade.

[30] Butko N.J., Movellan J.R. Optimal scanning for faster object detection. Proceedings of the 2009 IEEE Conference on Computer Vision and Pattern Recognition.

[31] Wenkel S., Alhazmi K., Liiv T., Alrshoud S., Simon M. (2021). Confidence Score: The Forgotten Dimension of Object Detection Performance Evaluation. Sensors.

[32] Zahid A., Mahmud M.S., He L., Heinemann P., Choi D., Schupp J. (2021). Technological advancements towards developing a robotic pruner for apple trees: A review. Comput. Electron. Agric..

